# Does the Type of Skin Marker Prevent Marking Erasure of Surgical-Site Markings?

**Published:** 2009-09-09

**Authors:** Simon C. Mears, Arman B. Davani, Stephen M. Belkoff

**Affiliations:** International Center for Orthopaedic Advancement, The Johns Hopkins University/Johns Hopkins Bayview Medical Center, Baltimore, Md.

## Abstract

**Objective:** Site marking is essential to prevent wrong-site surgery, and there are many skin markers commercially available. However, preoperative skin preparation can erase the site mark, especially when a chlorhexidine skin preparation solution that requires skin scrubbing is used. The purpose of our study was to test the hypothesis that some markers can withstand skin preparation with a chlorhexidine-based skin preparation solution in a manner similar to that of an iodine-based solution. **Methods:** On each of 5 cadaveric skin flaps, we made 2 rows of site markings with 9 types of markers. We then subjected one row of markings on each flap to a chlorhexidine-based solution and the other row to an iodine-based solution. A digital photograph was taken before and after each skin preparation. Using imaging software, the contrast in grayscale between the skin and skin marking was measured on each photograph. The effect of the type of marker and skin preparation solution on the difference in grayscale contrast was evaluated by multiple linear regression analysis and significant differences were determined (*P* < .05). **Results:** In all cases, the chlorhexidine-based skin preparation solution significantly decreased the contrast measured. No marker was significantly better than another. **Conclusions:** We conclude that all 9 skin markers are significantly erased with the chlorhexidine-based skin preparation solution. The development of a better skin marker or a chlorhexidine-based skin preparation solution that does not erase site markings is essential to prevent wrong-site surgeries and promote patient safety.

Site marking is an essential part of the preoperative process, and surgical associations currently mandate a time-out to verify the correct surgical site and surgery,^1^,^2^ which is meant to eliminate wrong-site surgery.^3^–^5^ The Joint Commission^6^ recommends that the site marking be within the surgical field after draping and that the time-out be performed just before surgical incision. Marking is also an essential part of surgical procedures such as mastopexy, in which the unanesthetized patient is marked preoperatively to provide optimal cosmesis and symmetry.^7^ The erasure of such site markings can cause problems in terms of site identification and surgical incision positioning.

Skin preparation is also part of the preoperative process, and chlorhexidine-based skin preparation solutions have been recommended as a way of decreasing the risk of postoperative wound infection.^8^,^9^ However, at least 1 commercially available chlorhexidine-based skin preparation solution (Chloraprep; chlorhexidine gluconate, 2% w/v, plus isopropyl alcohol, 70% v/v; Enturia, Inc, Leawood, Kan), which requires a surgical scrub application for 30 seconds, has been found to erase site marking when made with 1 type of marking pen.^10^ Although several other skin-marking pens (some disposable) are now available in different sizes and ink colors, to our knowledge, no study has assessed their ability to withstand such skin preparation.

Therefore, the purpose of this study was to test the hypothesis that some markers can withstand skin preparation with a chlorhexidine-based skin preparation solution in a manner similar to that with an iodine-based solution.

## METHODS

**Figure 1:**
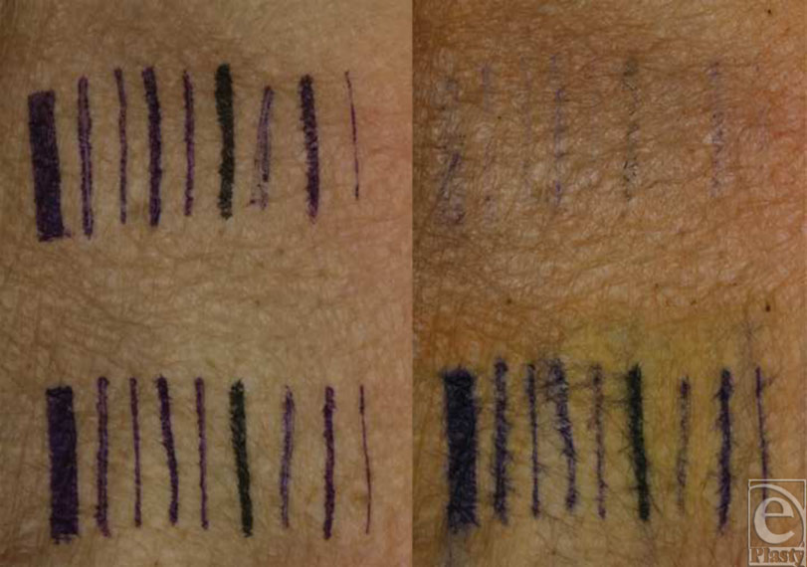
Photographs of skin markings before (left) and after (right) the application of a chlorhexidine-based (top) or iodine-based (bottom) skin preparation solution. Marks made with each of the pens from left to right are as follows: Sandel 4-in-1 marker, Waterproof Permanent Marker-Mini, OP-marks mini markers, OP-marks mini max, Accu-line wide body, Sharpie super permanent marker, Securline surgical skin marker no. 1000, HMS Twin-Tip broad, and HMS Twin-Tip fine.

Five flaps of skin from male white cadavers were obtained from the State Anatomy Board. The skin flaps were warmed to 20°C, and the temperature was measured with a thermocouple (K-type; Omega Engineering, Inc, Stamford, Conn). Nine commercially available pens specifically marketed for skin marking were identified through an Internet search. On each flap of skin, 2 separate rows of marks were made with each of the 9 types of pens: (1) Sandel 4-in-1 marker (skin, wide) (Sandel Medical Industries, LLC, Chatsworth, Calif); (2) Waterproof Permanent Marker-Mini, Fine Tip (Viscot Medical LLC, East Hanover, NJ); (3) OP-marks mini markers (OP-marks, Inc, Bogart, Ga); (4) OP-marks mini max (OP-marks, Inc); (5) Accu-line wide body (Accu-line Products, Inc, Hyannis, Mass); (6) Sharpie super permanent marker (Sanford Corporation, Oak Brook, Ill); (7) Securline surgical skin marker no. 1000 (Precision Dynamics Corporation, San Fernando, Calif); (8) HMS Twin-Tip broad (Hospital Marketing Services Co, Inc, Naugatuck, Conn); and (9) HMS Twin-Tip fine (Hospital Marketing Services Co, Inc). Each mark was a single vertical line that was approximately 50 mm long. Digital photographs (Fig 1) were obtained with a 10.1-megapixel camera (Digital Rebel XTi; Canon USA, Inc, Lake Success, NY), equipped with a 100-mm macro lens (EF 100-mm ƒ/2.8 USM Macro Lens; Canon USA, Inc) and a ring flash (MR-14EX TTL; Canon USA, Inc). Camera settings included a shutter speed of 1/60 seconds and an *F*-stop value of 4.0.

The markings were allowed to dry for at least 15 minutes before skin preparation. On each skin flap, a chlorhexidine-based skin preparation solution (Chloraprep) was applied to one row of markings and an iodine-based skin preparation solution (Duraprep; iodophor, 0.7% available iodine, plus isopropyl alcohol, 74% w/w; 3M Healthcare, St Paul, Minn), which served as the control, was applied to the other row. Both solutions were applied according to their respective manufacturer's guidelines. Chloraprep was applied for 30 seconds, with repeated forward and backward strokes of the applicator. Duraprep was applied by painting a single layer of the solution on the site. No scrubbing motion was used during Duraprep application. The surface of the skin flaps was allowed to dry completely after skin preparation and then we obtained a second set of digital images with the same camera and settings.

Adobe Photoshop CS2 (Adobe Systems, Inc, San Jose, Calif) was used to convert the raw digital images into a grayscale with 256 levels (with 0 being the darkest and 255 being white). The mean contrast in grayscale between each mark and its surrounding skin was determined with the program's histogram tool. The difference between grayscale contrast measurements of the images before and after skin preparation was calculated.

The effect of the type of marker and skin preparation solution on the difference in grayscale contrast was evaluated by multiple linear regression analysis (Stata10; StataCorp, LP, College Station, Tex). Unless otherwise specified, differences were considered significant at *P* < .05.

## RESULTS

The mean grayscale contrast for all markings with Chloraprep application was significantly lower than that of the preapplication marking (Table 1). Duraprep application did not significantly alter the mean grayscale contrast for any of the surgical markers (Table 1). There were no significant differences between markings subjected to Chloraprep, that is, none of the markings resisted erasure significantly better than another.

## DISCUSSION

Results of our study suggest that currently available skin markers are significantly erased when chlorhexidine-based skin preparation solution is used to prepare the skin, thus rejecting our hypothesis that there was a marker compatible with chlorhexidine-based skin preparation solution. We found no marker less vulnerable to erasure than another when exposed to the chlorhexidine-based skin preparation solution.

However, site-marking erasure might be prevented through various mechanisms. Marker technology could evolve so that the ink is more permanent and cannot be erased with skin preparation. The type of chlorhexidine preparation solution could be changed so that it does not erase the marks. It is possible that the recommended application method could be altered to lessen site-marking erasure. Currently, the chlorhexidine-based skin preparation solution is applied by a scrubbing technique because this is the method of application approved by the US Food and Drug Administration for this product. The scrubbing action, however, may exacerbate the erasure of the markings. Changing the skin preparation process would require US Food and Drug Administration approval and verification of effectiveness in skin decontamination.

The exact protocol of the time-out process could also be changed. We used the definition of the time-out process supplied by The Joint Commission Universal Protocol.^6^ With this method, the site marking must be visible in the prepared and draped surgical field immediately before skin incision. The time-out is then the final check before surgical incision.^3^ This strict definition is necessary to eliminate wrong-site surgery. Although it has been common practice to perform the time-out before positioning or skin preparation, doing so may introduce the opportunity for wrong-site surgery.

Limitations of this study include the use of cadaveric instead of live skin. It is unknown whether the 2 types of skin respond differently to marker ink. A randomized prospective study (ClinicalTrials.gov Identifier: NCT00739583) is currently underway to investigate the durability of surgical-site markings when exposed to skin preparation solutions. All specimens in the current study had light skin coloration (white). We expect to include skin with darker pigmentation, which may reduce the contrast of the ink and make site-marking visibility even more challenging. The threshold for marking erasure is also unknown. The surgeon subjectively determines the degree to which a surgical mark is not visible. Our position is the same as that of The Joint Commission; namely, that the marker used should make site markings sufficiently permanent to remain visible after completion of the skin preparation and sterile draping.^6^

In conclusion, we did not find any skin marker that withstood the effects of the chlorhexidine-based skin preparation solution. The grayscale contrast was significantly decreased for all types of markers after the application of Chloraprep. Additional effort is needed to ensure that surgical-site markings are retained when a chlorhexidine-based skin preparation solution is used.

## Figures and Tables

**Table 1:** Mean differences in grayscale contrast by skin preparation

		Mean (95% confidence interval) difference
Type of pen	Chloraprep	Duraprep
Sandel 4-in-1 marker (skin, wide)	40.5 (28.8–52.1)	4.4 (‐10.9 to 19.6)
Waterproof Permanent Marker-Mini, Fine Tip	47.8 (37.5–58.1)	3.9 (‐2.2 to 10.0)
OP-marks mini markers	47.9 (39.2–56.7)	3.7 (‐0.5 to 7.9)
OP-marks mini max	49.4 (39.5–59.4)	3.9 (‐4.2 to 12.0)
Accu-line wide body	51.2 (45.0–57.3)	9.9 (7.1–12.6)
Sharpie super permanent marker	53.5 (44.5–62.4)	9 (‐2.8 to 21.6)
Securline surgical skin marker no. 1000	36.0 (28.6–43.4)	12.3 (3.6–21.1)
HMS Twin-Tip broad	40.2 (33.5–47.0)	2.4 (0.0–4.8)
HMS Twin-Tip fine	26.2 (16.2–36.2)	2.4 (‐4.2 to 8.9)
